# Impact of preoperative TACE on incidences of microvascular invasion and long‐term post‐hepatectomy survival in hepatocellular carcinoma patients: A propensity score matching analysis

**DOI:** 10.1002/cam4.3814

**Published:** 2021-03-01

**Authors:** Yun Yang, Kongying Lin, Lei Liu, Youwen Qian, Yuan Yang, Shengxian Yuan, Peng Zhu, Jian Huang, Fuchen Liu, Fangming Gu, Siyuan Fu, Beige Jiang, Hui Liu, Zeya Pan, Wan Yee Lau, Weiping Zhou

**Affiliations:** ^1^ The Third Department of Hepatic Surgery Eastern Hepatobiliary Surgery Hospital Shanghai China; ^2^ Department of Hepatopancreatobiliary Surgery Mengchao Hepatobiliary Hospital of Fujian Medical University Fuzhou China; ^3^ The Department of Pathology Eastern Hepatobiliary Surgery Hospital Shanghai China; ^4^ Faculty of Medicine, the Chinese University of Hong Kong Hong Kong SAR China; ^5^ Key Laboratory of Signaling Regulation and Targeting Therapy of Liver Cancer (SMMU) Ministry of Education Shanghai China; ^6^ Shanghai Key Laboratory of Hepatobiliary Tumor Biology (EHBH Shanghai China

**Keywords:** hepatocellular carcinoma, microvascular invasion, prognosis, transcatheter arterial chemoembolization

## Abstract

**Background:**

To study the influence of preoperative transcatheter arterial chemoembolization (TACE) on the incidence of microvascular invasion (MVI) and long‐term survival outcomes in hepatocellular carcinoma (HCC) patients.

**Methods:**

Between January 1, 2010 and December 1, 2014, consecutive HCC patients who underwent curative liver resection were enrolled in this study. Univariable and multivariable regression analyses were used to identify independent predictive factors of MVI. Propensity score matching (PSM) was used to compare the incidences of MVI and prognosis between patients who did and did not receive preoperative TACE. Factors associated with Disease‐Free Survival (DFS) and Overall survival (OS) were identified using Cox regression analyses.

**Results:**

Of 1624 patients, 590 received preoperative TACE. The incidence of MVI was significantly lower in patients with preoperative TACE than those without preoperative TACE (39.15% vs. 45.36%, *p* = 0.015). After PSM, the incidences of MVI were similar in the two groups (38.85% vs. 41.10%, *p* = 0.473). Multivariable regression analysis revealed preoperative TACE to have no impact on the incidence of MVI. Before PSM, survival of patients with preoperative TACE was significantly worse than those without preoperative TACE (*p* = 0.032 for DFS and *p* = 0.027 for OS). After PSM, the difference became insignificant (*p* = 0.465 for DFS and *p* = 0.307 for OS). After adjustment for other prognostic variables in the propensity‐matched cohort, preoperative TACE was still found not to be associated with DFS and OS after HCC resection. Both before and after PSM, the prognosis of patients was not significantly different between the two groups for BCLC stages 0, A, and B.

**Conclusions:**

Preoperative TACE did not influence the incidence of MVI and prognosis of patients with HCC who underwent ‘curative’ liver resection.

## Graphical abstract

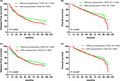

## 1 INTRODUCTION

Hepatocellular carcinoma (HCC) is one of the most prevalent malignant tumor worldwide. It is the fifth most commonly diagnosed malignant tumor and the third highest cause of cancer‐related death.^1^ Liver resection remains the mainstay of curative treatment for HCC. However, even for patients with early stage disease, recurrence and metastasis after surgery occur in approximately 50%–70% of patients.^2^, ^3^ Thus, measures to decrease tumor recurrence and prolong patient survival after surgery are urgently needed.

Previous studies have shown tumor size, tumor number, degree of tumor differentiation, AFP level, and surgical margins to be factors associated with postoperative recurrence.^4^, ^5^, ^6^ In addition, microvascular invasion (MVI) is a known high‐risk factor that leads to early recurrence after curative liver resection for HCC.^7^, ^8^, ^9^ Approximately 20%–60% of specimens obtained after liver resection for HCC are accompanied by MVI,^10^ which can also be found in early and even very early stages of HCC.^11^ Therefore, reducing MVI incidence is an effective method to improve the long‐term survival of HCC patients.

Our previous study has shown that the prognosis of patients with preoperative TACE was better than that of patients without preoperative TACE, although the difference was not significant.^12^ This may be associated with the different incidence of MVI between the two groups. In addition, no significant difference between the two groups in prognosis may attribute to relative small sample size in the study. Using a large HCC patient cohort, this study aimed to analyze the influence of preoperative TACE on the incidence of MVI and the long‐term post‐hepatectomy prognosis of patients. To reduce potential biases that are inherent in retrospective studies, propensity score matching (PSM) was used. PSM, a widely used statistical method in cohort studies, can match patients with similar distribution of confounders so that the difference in outcomes gives unbiased estimate of treatment effect.

## 2 METHODS AND PATIENTS

### 2.1 Methods

The study has been reported in line with the STROCSS criteria.^13^ The registration unique identifying number of this retrospective study is researchregistry6025. (https://www.researchregistry.com/browse‐the‐registry#home/).

### 2.2 Study population

In order to analyze the impact of preoperative TACE on the long‐term survival of HCC patients, consecutive HCC patients who underwent R0 resection at our Hospital between January 1, 2010 and December 1, 2014 were assessed to enter into this study. Preoperative diagnosis of HCC was based on the diagnostic criteria used by the American Association for the Study of Liver Diseases.^14^ The inclusion criteria for this study were patients with (a) no macroscopic vascular invasion or extrahepatic metastasis, (b) liver function of Child–Pugh A or selected B (score B 7), (c) age between 18–70 years, (d) complete serological data and underwent contrast‐enhanced computed tomography (CT) or magnetic resonance imaging (MRI), (e) R0 liver resection (the definition of R0 liver resection is complete removal of macroscopic tumor nodules and absence of microscopic tumor tissues at the operative margins),^15^ (f) a histopathological diagnosis of MVI determined by two experienced pathologists, (g) complete clinical and follow‐up data, and (h) no other anticancer treatment prior to surgery, including percutaneous ethanol injection, percutaneous radiofrequency ablation, chemotherapy, radiotherapy, molecular targeted agents, or portal vein embolization. The exclusion criteria were patients with (a) incomplete clinical and/or follow‐up data, (b) R1/R2 palliative resection, (c) HCC accompanied with other cancers, or (d) surgery‐related death within 30 days. The study was approved by the ethics committee of our hospital, and written informed consent was obtained from all participants for their data to be used in clinical research.

### 2.3 Preoperative TACE

TACE was performed as previously described.^12^ Briefly, a vascular catheter was inserted through a femoral artery using the Seldinger technique, and hepatic angiography was then performed. The catheter's tip was selectively inserted into the left or right hepatic artery, or the tumor‐feeding artery when technically possible. An emulsion of 5‐fluorouracil (1 g), mitomycin C (20 mg), cisplatin (5 mg), and lipiodol (10–30 ml; 1–2 ml/cm of tumor diameter) was then injected. Tumor feeding vessels were embolized with gelatin sponge.

All patients received routine blood tests, liver function, renal function, CEA, CA19‐9, and AFP 4–6 weeks after TACE. Radiographic responses were evaluated using the modified Response Evaluation Criteria in Solid Tumors (mRECISTs).^16^

### 2.4 Data collection

Routine preoperative examinations included imaging and serological examinations. All patients underwent abdominal ultrasonography, abdominal contrast‐enhanced MRI and/or CT scans, and chest X‐ray or chest CT scans. All radiological data were reviewed using unified diagnostic criteria by two independent radiologists who had >10 years of radiological experience. Preoperative laboratory tests included routine blood tests, liver function, renal function, coagulation profile, hepatitis B surface antigen/e antigen, hepatitis C antibody, hepatitis B virus DNA load (HBV DNA), hepatitis C virus RNA load, levels of alpha‐fetoprotein (AFP), carcinoembryonic antigen (CEA), and carbohydrate antigen 19–9 (CA19‐9). HBV DNA, AFP, total bilirubin (TBIL), albumin (ALB), alanine aminotransferase (ALT), and platelet (PLT) were coded as binary variables according to the cutoff points that were reported previously.^17^ Esophageal and gastric varices were diagnosed by esophagogastroduodenoscopy. The Barcelona Clinic Liver Cancer (BCLC) staging classification was used for tumor staging.^18^

### 2.5 Preoperative evaluations and surgical procedures

Resectability of HCC was evaluated according to patient's general condition, liver function, tumor size, tumor location, and amount of future remnant liver volume, as estimated based on CT and/or MRI scans before operation. The technique used for R0 liver resections was as previously described.^19^ Satellite nodules were defined as tumors <1 cm in diameter and located <1 cm from the main tumor. Currently, the definition of MVI is the presence of tumor cells in a portal vein, hepatic vein, or large capsular vessel of the surrounding hepatic tissue lined by endothelium that is only visible under microscopy.^9^ All histopathological evaluations were performed independently by two pathologists with >10 years of experience. The two pathologists were blinded to all clinical data.

### 2.6 Follow‐up and endpoints

All patients were reviewed once every 3 months after surgery. Postoperative follow‐up was performed by the same team of surgeons, and the follow‐up program included serum AFP, complete blood counts, liver function, renal function, hepatitis B virus DNA, hepatitis C virus RNA, chest X‐ray, abdominal B‐ultrasound, and abdominal contrast‐enhanced CT or MRI. When a patient was diagnosed with tumor recurrence, appropriate treatments, such as percutaneous ethanol injection, radiofrequency ablation, TACE, or re‐hepatectomy, were performed based on the patient's general condition, liver function, tumor size, pattern of tumor recurrence, and patient's wishes. Treatment of extrahepatic metastasis included local excision, systemic chemotherapy, and molecular targeted therapy. The best supportive care was given to patients with end‐stage disease, poor liver function, or poor general status. This study was censored on June 1, 2019.

The median (interquartile range) duration of follow‐up was 48 (22–86) months. The primary endpoints were overall survival (OS), which was measured from the date of surgery to the date of patient death or last follow‐up, and time to recurrence, which was calculated from the date of surgery to the date when tumor recurrence was diagnosed. The secondary outcome was the presence of MVI based on postoperative histopathology.

### 2.7 Propensity score matching (PSM)

Patients with preoperative TACE were matched with patients without preoperative TACE using the PSM as previous description. ^20^, ^21^, ^22^ Covariates entered into the PSM model included gender, age, sex, HbsAg, HbeAg, HCV Ab, HBV‐DNA load, AFP levels, TBIL, ALB, ALT, preoperative platelets count, tumor number, cirrhosis, maximum tumor size, tumor encapsulation, tumor margin, tumor differentiation, and satellite nodules. PSM was performed as a 1:1 matching between the two groups, with nearest‐neighbor matching and a 0.05 caliper width. The matching procedure has been described previously.^23^

### 2.8 Statistical analysis

Continuous variables were expressed as median (range) or mean (SD). Categorical variables were expressed as frequency (percentage). Categorical variables were compared by the χ2 test or Fisher's exact test. Continuous variables were compared by the student's t test or Mann–Whitney U test. All analyses were two‐tailed. Survival curves of patients with and without preoperative TACE before and after propensity matching were calculated using the Kaplan–Meier method and compared using the log‐rank test. Preoperative factors that might be associated with MVI presence were identified by univariable and multivariable regression analyses. The Cox proportional hazard regression model was used to adjust for other prognostic factors, which were associated with DFS and OS. All statistical analyses were performed using SPSS 23.0 for Windows (SPSS, IBM, Armonk, NY, USA). A *p* value <0.05 was considered statistically significant.

## 3 RESULTS

### 3.1 Patient clinical characteristics

During the study period, of 2532 patients who underwent R0 liver resection for HCC, 612 received and 1920 patients did not receive preoperative TACE. The reasons why these 612 patients decided to receive TACE as the first treatment were because of personal/medical reasons to delay major surgery (*n* = 230), initial refusal to surgical treatment (*n* = 225), or socio‐financial reasons (*n* = 157). Figure [Link], [Link] shows the reasons why 908 patients from the whole cohort of 2532 patients were excluded from this study. Finally, 1624 patients were enrolled into this study.

Of 1624 patients who were included in this study, 590 received preoperative TACE, while the remaining 1034 did not receive preoperative TACE. Among the 590 patients who were treated with preoperative TACE, 438 received a single session of preoperative TACE and 152 received multiple sessions. The median number of preoperative TACE sessions was 1 (range: 1–6). The median interval between the first TACE treatment and surgery was 11 weeks (range: 2–42). For patients with multiple preoperative TACE sessions, the median interval between the last TACE treatment and surgery was 8 weeks (range: 2–29).

Baseline characteristics of the study cohorts are listed in Table 1. There were no significant differences in serum AFP levels, percentages of HBsAg(+), HBeAg(+), and HCV Ab(+), or degrees of tumor differentiation between the two groups (*p* > 0.05). Patients in the preoperative TACE group were significantly younger (*p* < 0.05), and the proportion of male patients was significantly higher (*p* < 0.05). The HBV DNA load was significantly lower in patients who received preoperative TACE than those who did not receive preoperative TACE. There were significantly higher total bilirubin (TBIL) and alanine aminotransferase (ALT) levels and a higher percentage of accompanying liver cirrhosis in patients with preoperative TACE (*p* < 0.001), while the albumin and platelet levels in the preoperative TACE group were significantly lower than in patients without preoperative TACE. Patients with preoperative TACE had significantly larger tumor sizes (*p* < 0.001), and a significantly higher proportion had multiple tumors (*p* < 0.001). The percentages of smooth tumor margins and complete tumor capsules were significantly higher, while the proportion of satellite lesions was significantly lower in the preoperative TACE group (*p* < 0.05). PSM analysis created 489 pairs of patients. Comparisons of patients’ baseline characteristics between the two groups in the propensity matched cohort are illustrated in Table 1. After PSM, there were no significant differences in the background characteristics or preoperative factors between the two groups (*p* > 0.05).

**TABLE 1:** Comparisons of patients’ baseline characteristics between patients with and without preoperative transarterial chemoembolization (TACE) before and after propensity score matching (PSM)

The entire cohort				The PSM cohort			
Variables	With preoperative TACE (*N* = 590)	Without preoperative TACE (*N* = 1034)		variables	With preoperative TACE (*N* = 489)	Without preoperative TACE (*N* = 489)	
	*N* (%)	*N* (%)	*p*		*N* (%)	*N* (%)	*p*
Age, years (Mean±SD)	50.21 ± 10.40	51.95 ± 10.22	0.001	Age, years (Mean±SD)	50.82 ± 10.31	51.40 ± 10.21	0.378
Gender			0.021	Gender			0.139
Male	520(88.14)	868(83.95)		Male	429(87.73)	413(84.46)	
Female	70(11.86)	166(16.05)		Female	60(12.27)	76(15.54)	
HbsAg			0.124	HbsAg			0.579
+	512(86.78)	868(83.95)		+	418(85.48)	420(85.89)	
−	78(13.22)	166(16.05)		−	71(14.52)	69(14.11)	
HbeAg			0.322	HbeAg			0.269
+	161(27.29)	259(25.05)		+	130(26.58)	115(23.52)	
−	429(72.71)	775(74.95)		−	359(73.42)	374(76.48)	
HCV Ab			0.263	HCV Ab			0.634
+	13(2.20)	15(1.45)		+	10(2.04)	8(1.64)	
−	577(97.80)	1019(98.55)		−	479(97.96)	481(98.36)	
HBV DNA			<0.001	HBV DNA			0.176
≥10,000 IU/ml	144(24.41)	351(33.95)		≥10,000 IU/ml	127(25.97)	146(29.86)	
<10,000 IU/ml	446(75.59)	683(66.05)		<10,000 IU/ml	362(74.03)	343(70.14)	
AFP			0.128	AFP			0.552
≥400 ng/ml	230(38.98)	364(35.20)		≥400 ng/ml	187(38.24)	178(36.40)	
<400 ng/ml	360(61.02)	670(67.80)		<400 ng/ml	302(61.76)	311(63.60)	
TBIL			<0.001	TBIL			0.812
≥17umol/L	162(27.46)	142(13.73)		≥17umol/L	101(20.65)	98(20.04)	
<17umol/L	428(72.54)	892(86.27)		<17umol/L	388(79.35)	391(79.96)	
ALB			<0.001	ALB			0.896
≥35 g/L	319(54.07)	714(69.05)		≥35 g/L	294(60.12)	292(59.71)	
<35 g/L	271(45.93)	320(30.95)		<35 g/L	195(39.88)	197(40.29)	
ALT			<0.001	ALT			0.653
≥44 U/L	281(47.63)	387(37.43)		≥44 U/L	214(43.76)	221(45.19)	
<44 U/L	309(52.37)	647(62.57)		<44 U/L	275(56.24)	268(54.81)	
PLT			<0.001	PLT			1.000
≥100*10^9/L	434(73.56)	859(83.08)		≥100*10^9/L	371(75.87)	371(75.87)	
<100*10^9/L	156(26.44)	175(16.92)		<100*10^9/L	118(24.13)	118(24.13)	
Tumor number			<0.001	Tumor number			0.764
Sinle	426(72.20)	849(82.11)		Sinle	370(75.66)	374(76.48)	
Multiple	164(27.80)	185(17.89)		Multiple	119(23.34)	115(23.52)	
Liver Cirrhosis			<0.001	Liver Cirrhosis			0.168
Yes	273(46.27)	283(27.37)		Yes	200(40.90)	179(36.61)	
No	317(53.73)	751(72.63)		No	289(59.10)	310(63.39)	
Max Tumor diameter(Mean±SD)	7.28 ± 4.68 cm	6.42 ± 4.20 cm	<0.001	Max Tumor diameter(Mean±SD)	6.97 ± 4.43 cm	6.76 ± 4.42 cm	0.445
Tumor capsule				Tumor capsule			0.228
Absent or Partial	463(78.74)	885(85.59)	<0.001	Absent or Partial	400(81.80)	385(78.73)	
Complete	127(21.53)	149(14.41)		Complete	89(18.20)	104(21.27)	
Tumor margin			0.002	Tumor margin			0.749
Smooth	479(81.19)	771(74.56)		Smooth	393(80.37)	389(79.55)	
Non‐smooth	111(18.81)	263(25.43)		Non‐smooth	96(19.63)	100(20.45)	
Edmondson Grade			0.995	Edmondson Grade			0.413
I+II	61(10.33)	115(11.12)		I+II	57(11.66)	66(13.50)	
III+IV	487(82.54)	919(88.88)		III+IV	428(87.53)	423(86.50)	
Satellite Nodules			0.021	Satellite Nodules			0.798
Presence	273(46.27)	540(52.22)		Presence	237(48.47)	241(49.28)	
Absence	317(53.73)	494(47.78)		Absence	252(51.53)	248(50.72)	

Abbreviations: AFP, serum alpha‐fetoprotein; ALB, albumin; ALT, alanine aminotransferase; DNA, deoxyribonucleic acid; HBeAg, hepatitis B e antigen; HBsAg, hepatitis B surface antigen; HBV, hepatitis B virus; HCV Ab, hepatitis C virus antibody; PLT, platelet; PSM, Propensity score matching; TACE, transcatheter arterial chemoembolization; TBIL, total bilirubin.

The 1624 patients were then stratified according to the BCLC classification: 79 patients were classified into stage 0, 1111 were classified into stage A, and 434 were classified into stage B. For patients in stage 0, platelet levels in the patients with preoperative TACE were significantly lower than in patients without preoperative TACE (*p < *0.001). The proportion of patients with liver cirrhosis and complete tumor capsules of preoperative TACE group were significantly higher than that of non‐preoperative TACE group (*p < *0.05). The other clinical characteristics between the two groups were not different significantly (Table S1). PSM analysis was not performed further because of small sample size.

For patients in stage A, 364 patients received preoperative TACE and 747 patients did not receive preoperative TACE. Patients with preoperative TACE were younger than patients without preoperative TACE (*p < *0.05). HBV DNA load, ALB, PLT, and proportion of satellite lesions were significantly lower in the preoperative TACE group than that in the non‐preoperative TACE group (*p < *0.05), while TBIL, ALT, percentage of accompanying liver cirrhosis, and proportion of smooth tumor margins and complete tumor capsules were significantly higher in the preoperative TACE group than the non‐preoperative group (*p < *0.05). In addition, patients with preoperative TACE had significantly larger tumor sizes (*p* < 0.001) than patients without preoperative TACE. After PSM, there were 315 patients in the preoperative TACE group and non‐preoperative group, respectively. The baseline characteristics between the two groups were not different significantly (*p* > 0.05) (Table S2).

For patients in stage B, 194 patients received preoperative TACE and 240 patients did not receive preoperative TACE. Patients in the preoperative TACE group had significantly higher TBIL than patients in the non‐preoperative TACE group (*p < *0.05). Additionally, the proportion of accompanying liver cirrhosis and complete tumor capsules was significantly higher in the preoperative TACE group than the non‐preoperative group (*p < *0.05). After PSM, there were no significant differences in baseline characteristics between the two groups (*p* > 0.05) (Table S3).

### 3.2 Tumor recurrence and overall survival (OS) between patients with and without preoperative TACE

**FIGURE 1:**
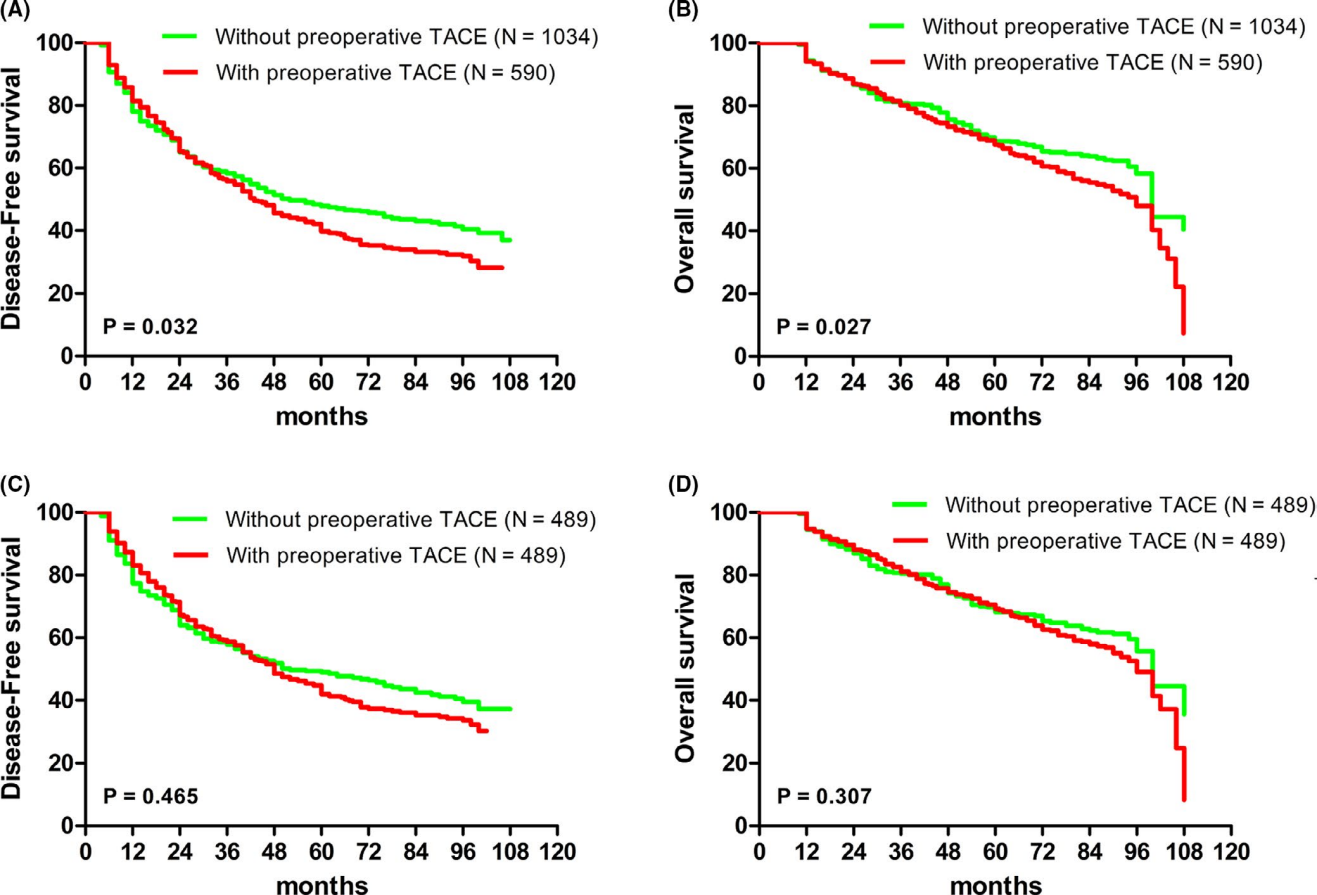
Survival analysis of patients with and without preoperative transarterial chemoembolization (TACE) in the entire cohort. (A) The cumulative Disease‐free survival (DFS) curve of HCC patients with preoperative TACE and patients without preoperative TACE before PSM (*p* = 0.032). (B) The cumulative overall survival (OS) curve of HCC patients with preoperative TACE and patients without preoperative TACE before PSM (*p* = 0.027). (C) The cumulative DFS curve of HCC patients with preoperative TACE and patients without preoperative TACE after PSM (*p* = 0.465). (D) The cumulative OS curve of HCC patients with preoperative TACE and patients without preoperative TACE after PSM (*p* = 0.307)

The 1‐, 3‐, and 5‐year disease‐free survival (DFS) rates of the preoperative TACE group were 80.33%, 55.21%, and 39.41%, respectively, compared with the without preoperative TACE group of 78.24%, 58.34%, and 47.90%, respectively. The cumulative DFS in patients with preoperative TACE was significantly lower than that in patients without preoperative TACE (*p* = 0.032). The 1‐, 3‐, and 5‐year cumulative OS rates of the preoperative TACE group were 93.89%, 72.82%, and 62.23%, respectively, compared with the without preoperative TACE group of 94.48%, 80.81%, and 68.62%, respectively. Thus, the OS rates of patients with preoperative TACE were significantly lower than those without preoperative TACE (*p* = 0.027) (Figure 1A and B). After PSM, the 1‐, 3‐, and 5‐year DFS rates in the preoperative TACE group were 82.20%, 57.53%, and 41.05%, respectively, while the corresponding figures for the without preoperative TACE group were 77.71%, 57.92%, and 48.93%, respectively. There was no significant difference between the two groups after PSM (*p* = 0.465). After PSM, the 1‐, 3‐, and 5‐year cumulative OS rates of the preoperative TACE group were 94.72%, 82.62%, and 67.73%, respectively, compared with the without preoperative TACE of 94.52%, 80.43%, and 68.27%, respectively. The difference in OS between the two groups after PSM was not significant (*p* = 0.307) (Figure 1C and D).

For patients in stage 0, the prognosis of patients with preoperative TACE was similar to those without preoperative TACE (1‐, 3‐, and 5‐year DFS rates of 84.38%, 78.13%, and 65.63% vs. 89.36%, 76.60%, and 68.09%, respectively *p* = 0.729). Similarly, the 1‐, 3‐, and 5‐year OS rates in those with and without preoperative TACE were 100%, 90.63%, and 84.38% versus 97.87%, 93.62%, and 91.49%, respectively (*p* = 0.445) (Figure S2A and B).

For patients in stage A, 364 received preoperative TACE, and their prognosis was worse, though insignificantly, than those without preoperative TACE (*n* = 747). The 1‐, 3‐, and 5‐year DFS rates in stage A patients with and without TACE were 85.71%, 64.01%, and 47.82% versus 82.46%, 65.32%, and 54.52%, respectively (*p* = 0.084). The 1‐, 3‐, and 5‐year OS rates of patients with and without TACE were 95.33%, 85.91%, and 72.27% versus 95.98%, 85.68%, and 75.66%, respectively (*p* = 0.052) (Figure S3A and B). After PSM, the respective 1‐, 3‐, and 5‐year DFS rates in patients with preoperative TACE were 86.35%, 65.18%, and 48.02%, while those in patients without preoperative TACE were 78.85%, 62.83%, and 52.95%, respectively. The difference was not significant (*p* = 0.819). The 1‐, 3‐, and 5‐year OS rates were 95.87%, 86.50%, and 72.56%, respectively, in patients with preoperative TACE and 95.46%, 84.35%, and 74.1%, respectively, in patients without preoperative TACE. Thus, there was no significant difference in the OS rates between the two groups (*p* = 0.399) (Figure S3C and D).

In BCLC stage B patients, the prognosis of the preoperative TACE group was similar to that of the non‐preoperative TACE group; the 1‐, 3‐, and 5‐year DFS rates were 69.59%, 40.20%, and 25.35% versus 62.52%, 33.18%, and 23.88%, respectively (*p* = 0.938). Similarly, the 1‐, 3‐, and 5‐year OS rates were 90.72%, 65.82%, and 54.73%, versus 88.96%, 63.12%, and 42.05%, respectively (*p* = 0.736) (Figure S4A and B). After PSM, the 1‐, 3‐, and 5‐year DFS rates in patients with and without preoperative TACE were 69.39%, 37.72%, and 28.82% versus 63.95%, 36.92%, and 29.26%; the difference between the two groups was not significant (*p* = 0.540). After PSM, the 1‐, 3‐, and 5‐year OS rates were 89.72%, 65.22%, and 52.42%, respectively, in patients with preoperative TACE and 89.80%, 62.42%, and 42.33%, respectively, in patients without preoperative TACE. The differences in OS rates between the two groups were not significant (*p* = 0.676) (Figure S4C and D).

### 3.3 Tumor recurrence and overall survival (OS) between patients with different intervals between the first preoperative TACE and liver resection

According to the intervals between the first TACE treatment and surgery, 590 patients with preoperative TACE were divided into two groups: intervals ≤4 weeks (*n* = 76) and >4 weeks (*n* = 514). The 1‐, 3‐, and 5‐year disease‐free survival (DFS) rates of patients with intervals ≤4 weeks were 73.51%, 45.42%, and 30.71%, respectively, compared with those in patients with intervals >4 weeks of 82.51%, 57.44%, and 41.22%, respectively. The cumulative DFS in patients with intervals ≤4 weeks was significantly lower than that in patients with intervals >4 weeks (*p* = 0.031). The 1‐, 3‐, and 5‐year cumulative overall survival (OS) rates of the group of patients with intervals ≤4 weeks were 94.71%, 77.12%, and 62.53%, respectively, compared with those patients with intervals >4 weeks of 94.02%, 80.52%, and 68.34%, respectively. Thus, the OS rates of patients with intervals ≤4 weeks were significantly lower than those with intervals >4 weeks (*p* = 0.044) (Figure S5A and B).

### 3.4 Association between preoperative TACE and incidences of MVI

The proportion of patients with MVI was significantly lower in the preoperative TACE group (39.15% vs. 45.36%, *p* = 0.015). The difference in the incidences of MVI between the two groups after PSM also became insignificant for the entire patient cohort (38.85% vs. 41.10%, *p* = 0.473) (Table 2). On subgroup analysis after stratification by the BCLC staging system, there were no significant differences in MVI incidences between patients with and without preoperative TACE in patients in BCLC stage 0 (15.63% vs. 25.53%, *p* = 0.296) (Table 2), but with significant differences in patients in BCLC stage A, (29.95% vs. 39.49%, *p* = 0.002). After PSM, the differences between the two groups became insignificant (31.43% vs. 35.56%, *p* = 0.273) (Table 2). In patients in BCLC stage B, there were no significant differences in MVI incidences between the two groups either before or after PSM (before PSM: 60.31% vs. 67.50%, *p* = 0.121; after PSM: 61.22% vs. 59.18%, *p* = 0.721) (Table 2).

**TABLE 2:** Microvascular invasion (MVI) incidences between patients with preoperative transarterial chemoembolization (TACE) and without preoperative transarterial chemoembolization (TACE) before and after propensity score matching (PSM)

	The entire cohort			The PSM cohort		
			*p*			*p*
All patients	With preoperative TACE (*N* = 590)	Without preoperative TACE (*N* = 1034)		With preoperative TACE (*N* = 489)	Without preoperative TACE (*N* = 489)	
	231/590(39.15%)	469/1034(45.36%)	0.015	190/489(38.85%)	201/489(41.10%)	0.473
Patients in BCLC Stage 0	With preoperative TACE (*N* = 32)	Without preoperative TACE (*N* = 47)		—	—	
	5/32(15.63%)	12/47(25.53%)	0.296	—	—	—
Patients in BCLC Stage A	With preoperative TACE (*N* = 364)	Without preoperative TACE (*N* = 747)		With preoperative TACE (*N* = 315)	Without preoperative TACE (*N* = 315)	
	109/364(29.95%)	295/747(39.49%)	0.002	99/315(31.43%)	112/315(35.56%)	0.273
Patients in BCLC Stage B	With preoperative TACE (*N* = 194)	Without preoperative TACE (*N* = 240)		With preoperative TACE (*N* = 147)	Without preoperative TACE (*N* = 147)	
	117/194(60.31%)	162/240(67.50%)	0.121	90/147(61.22%)	87/147(59.18%)	0.721

Abbreviations: BCLC, Barcelona Clinic Liver Cancer; MVI, Microvascular invasion; PSM, propensity score matching; TACE, transcatheter arterial chemoembolization.

### 3.5 Association of preoperative TACE with presence of MVI

Further analyses were conducted on the entire patient cohort who did and did not receive preoperative TACE (*n* = 1624). Multivariable regression analysis indicated that large tumor size, satellite nodules, grade III/IV differentiation, non‐smooth tumor margins, and high α‐fetoprotein levels were independently associated with increased risks of MVI. In this entire cohort, preoperative TACE had no impact on the incidence of MVI (Table 3).

**TABLE 3:** Logistic regression analysis of microvascular invasion presence in patients receiving preoperative TACE treatment and those not receiving preoperative TACE treatment (*N* = 1624)

Variable	Univariate		Multivariate	
	OR (95% CI)	*p*	OR (95% CI)	*p*
Preoperative TACE (Yes vs. No)	0.775(0.631–0.952)	0.015	0.848(0.660–1.090)	0.198
Tumor number (Multiple vs. Single)	1.569(1.237–1.991)	<0.001	0.795(0.583–1.083)	0.146
Tumor size (≥5 cm vs. <5 cm)	1.980(1.619–2.422)	<0.001	1.759(1.375–2.250)	<0.001
Satellite nodules (Presense vs. Absense)	5.268(4.245–6.538)	<0.001	3.397(2.583–4.467)	<0.001
Edmondson grade (III+IV vs. I+II)	4.592(3.04–6.920)	<0.001	3.457(2.163–5.528)	<0.001
Tumor capsule (Non‐complete vs. Complete)	3.029(2.242–4.092)	<0.001	1.185(0.835–1.682)	0.341
Liver cirrhosis (Yes vs. No)	1.110(0.902–1.364)	0.324	—	—
Age (≥60 vs. <60)	0.835(0.664–1.050)	0.123	—	—
Gender (Male vs. Female)	1.195(0.902–1.584)	0.215	—	—
Tumor margin (Non‐smooth vs. Smooth)	12.903(9.478–17.566)	<0.001	8.874(6.357–12.387)	<0.001
HCV Ab (Positive vs. Negative)	0.852(0.396–1.830)	0.681	—	—
HBV DNA (≥10,000 IU/ml VS. <10,000 IU/ml)	0.950(0.767–1.176)	0.635	—	—
TBIL (≥17µmol/L vs. <17µmol/L)	0.861(0.668–1.109)	0.246	—	—
ALT (≥44 U/L vs. <44 U/L)	1.001(0.820–1.222)	0.994	—	—
ALB (<35 g/L vs. ≥35 g/L)	0.833(0.679–1.023)	0.091	—	—
PLT (<100*10^9/L vs. ≥100*10^9/L	0.945(0.740–1.206)	0.648	—	—
AFP (≥400 ng/ml vs. <400 ng/ml)	1.998(1.627–2.452)	<0.001	1.459(1.138–1.872)	0.003
HbeAg (Positive vs. Negative)	1.040(0.831–1.301)	0.734	—	—
HbsAg (Positive vs. Negative)	1.249(0.945–1.651)	0.118	—	—

Abbreviations: 95% CI, 95 Percent confidence interval; AFP, serum alpha‐fetoprotein; ALB, albumin; ALT, alanine aminotransferase; DNA, deoxyribonucleic acid; HBeAg, hepatitis B e antigen; HBsAg, hepatitis B surface antigen; HBV, hepatitis B virus; HCV Ab, hepatitis C virus antibody; OR, odds ratio; PLT, platelet; TACE, transcatheter arterial chemoembolization; TBIL, total bilirubin.

### 3.6 Independent risk factors for DFS and OS in HCC patients in the entire cohort and PSM cohort

The results of univariable and multivariable analysis of DFS and OS in the entire cohort are shown in Table S4 and Table S5. Univariable analysis suggested that preoperative TACE was associated with both DFS and OS, however, on multivariable analysis, preoperative TACE was not independently associated with both DFS and OS. In the PSM cohort, the results of univariable and multivariable analysis of DFS and OS are presented in Table S6 and Table S7. Preoperative TACE had no impact on both DFS and OS in the PSM cohort.

## 4 DISCUSSION

Postoperative recurrences of HCC, especially early recurrences, are closely related to small metastases that cannot be found by preoperative imaging,^12^ and MVI leads to recurrence of these small metastases. Thus, measures that can reduce the incidence of MVI are of great significance to reduce the rate of recurrence and to prolong post‐hepatectomy survival of HCC patients. This study indicated that preoperative TACE did not reduce the incidence of MVI and improve the prognosis of HCC patients after liver resection.

Previous studies have shown that the tumor size, tumor number, tumor capsule, tumor margins, AFP level, degree of tumor enhancement in arterial phase on intravenous enhanced CT, platelet number, and HBV level are closely related to occurrence of MVI.^11^, ^24^, ^25^, ^26^, ^27^ The influence of preoperative TACE on MVI occurrence is still unclear. Li et al. reported that the incidence of MVI was significantly lower in patients with huge HCC (≥10 cm) who had preoperative TACE when compared with patients without preoperative TACE.^28^ An alternative explanation for this difference can be in the differences in AFP levels in the two groups of patients. In that study, the AFP level in patients with preoperative TACE was significantly lower than those in patients without preoperative TACE, and AFP is a known independent risk factor for occurrence of MVI.^11^, ^24^, ^26^ Other studies have indicated that preoperative TACE can promote formation of a tumor capsule,^29^ and the incidence of MVI in patients with a complete capsule is significantly lower than in patients with an incomplete or without a capsule.^25^, ^27^ In the present study, the proportion of patients with a complete tumor capsule and smooth tumor boundaries were significantly higher in patients who received preoperative TACE. Furthermore, the HBV DNA level in the preoperative TACE group in the study was significantly lower than in patients without preoperative TACE. These factors can account for the significantly lower MVI incidence in patients with preoperative TACE than in patients without preoperative TACE. After PSM, there were no significant differences in patient characteristics, tumor size, tumor capsule, tumor boundary, and HBV DNA quantity between the two groups, and the analysis also showed no significant difference in MVI incidences between the two groups. When patients were classified using the BCLC Staging System, there were no significant differences in the incidences of MVI between the two groups, regardless of whether the patients were in stage 0, stage A, or stage B. In addition, multivariable regression analysis revealed that there is no impact of preoperative TACE on the incidence of MVI.

The impact of preoperative TACE on prognosis of HCC patients has been reported, but with controversial results. Some studies showed that prognosis after preoperative TACE was worse than that in patients without preoperative TACE,^30^, ^31^ whereas, others reported preoperative TACE to improve prognosis in patients with huge HCC (≥10 cm) or with portal vein tumor thrombus.^28^, ^32^, ^33^ Our previous randomized controlled trial showed that preoperative TACE did not improve prognosis in patients with a large but resectable HCC.^12^ It is now generally accepted that patients with resectable HCC should undergo surgery without preoperative TACE unless there is a good reason to delay surgery, e.g., to stabilize patient's associated medical conditions, initial refusal to surgery, or because of personal reasons. The sample of our randomized study was too small to perform further analyses of subgroups. In this study, PSM was used to adjust for potential confounding factors and to reduce selection bias between the two groups. Survival analyses indicated that there was no significant difference in long‐term DFS and OS between patients with and without perioperative TACE. When the patients were classified using the BCLC Staging System, there were no significant differences in the long‐term DFS and OS between the two groups, regardless of whether the patients were in stage 0, stage A, or stage B. Multivariable Cox analysis of DFS and OS suggested that preoperative TACE was not independently associated with either DFS or OS in the entire HCC or the PSM cohorts. Previous studies have shown that prognosis of patients with tumor necrosis area >90% to be significantly better than patients with tumor necrosis area ≤90%.^34^ Also, long term survival outcomes of patients with incomplete or no tumor necrosis after TACE were significantly worse after hepatic resection in patients with preoperative TACE than in patients without preoperative TACE.^35^ Furthermore, multivariate cox analysis showed that tumor necrosis area >90% to be an independent protective factor of recurrence and survival.^34^ A longer time between the first TACE procedure and liver resection has been reported to be related to the tumor necrosis area >90%.^36^ In this study, the impact of different intervals between the first TACE and liver surgery on prognosis of HCC patients was also analyzed. Survival of patients with intervals ≤4 weeks was significantly worse than that in patients with intervals >4 weeks. A possible explanation is that iodized oil needs a long period to exert its effect as the deposited amount of iodized oil has been demonstrated not to correlate with tumor necrosis within 20 days. However, after 20 days, there was an obvious correlation between them, and a study reported that tumor necrosis was mainly due to long‐term deposition of iodized oil.^37^ So tumor response to preoperation TACE affects prognosis of HCC patients. The criteria in selecting patients who can benefit from preoperative TACE need to be further studied.

### 4.1 Limitations

This study had several limitations. First, this is a single institutional retrospective study with its own inherent defects. Second, the majority of patients in this study had HBV‐related HCC. Patients with HCV‐related HCC and those with other etiologies will need to be further studied to support the findings of this study.

## CONFLICT OF INTEREST

No potential conflicts of interest were disclosed.

## ETHICAL APPROVAL AND INFORMED CONSENT

The study was approved by the Institutional Ethics Committee of the EHBH. Informed consent was obtained from all the patients for their data to be used for research.

## Supporting information

Figure S1

Figure S2

Figure S3

Figure S4

Figure S5

Table S1

Table S2

Table S3

Table S4

Table S5

Table S6

Table S7

Table S7

## Data Availability

All related data of our center are stored in the National Liver Tissue Bank (NLTB, www.nltb.org). The data that support the findings of this study are available from the National Liver Tissue Bank (NLTB, www.nltb.org), but restrictions apply to the availability of these data, which were used under license for the current study, and so are not publicly available. However, all related data in this study are available from the corresponding author on reasonable request.
