# Supplementary figures and images for: Impact of preoperative TACE on incidences of microvascular invasion and long‐term post‐hepatectomy survival in hepatocellular carcinoma patients: A propensity score matching analysis
Source: Cancer Med. 2021 Mar 1;10(6):2100–11. doi: 10.1002/cam4.3814 (PMC7957201; doi:10.1002/cam4.3814)

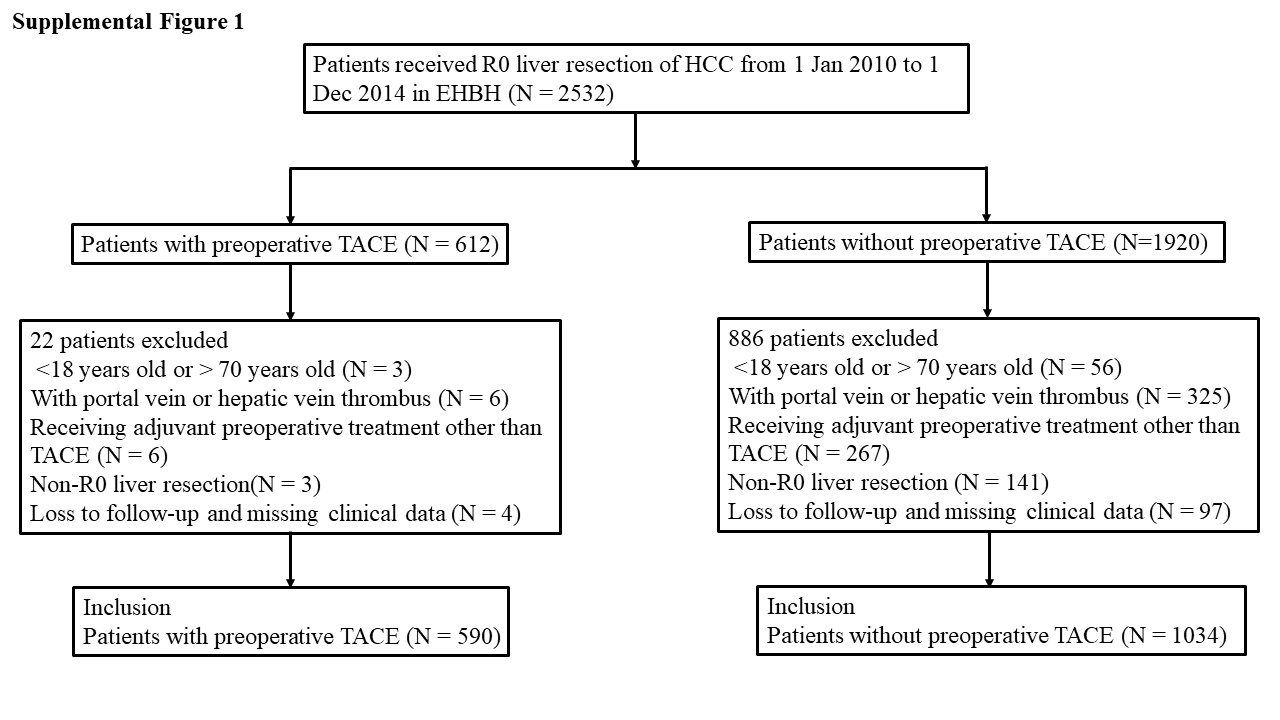

Supplement: Supplementary file 1 — Figure S1 [file CAM4-10-2100-s009.tif]

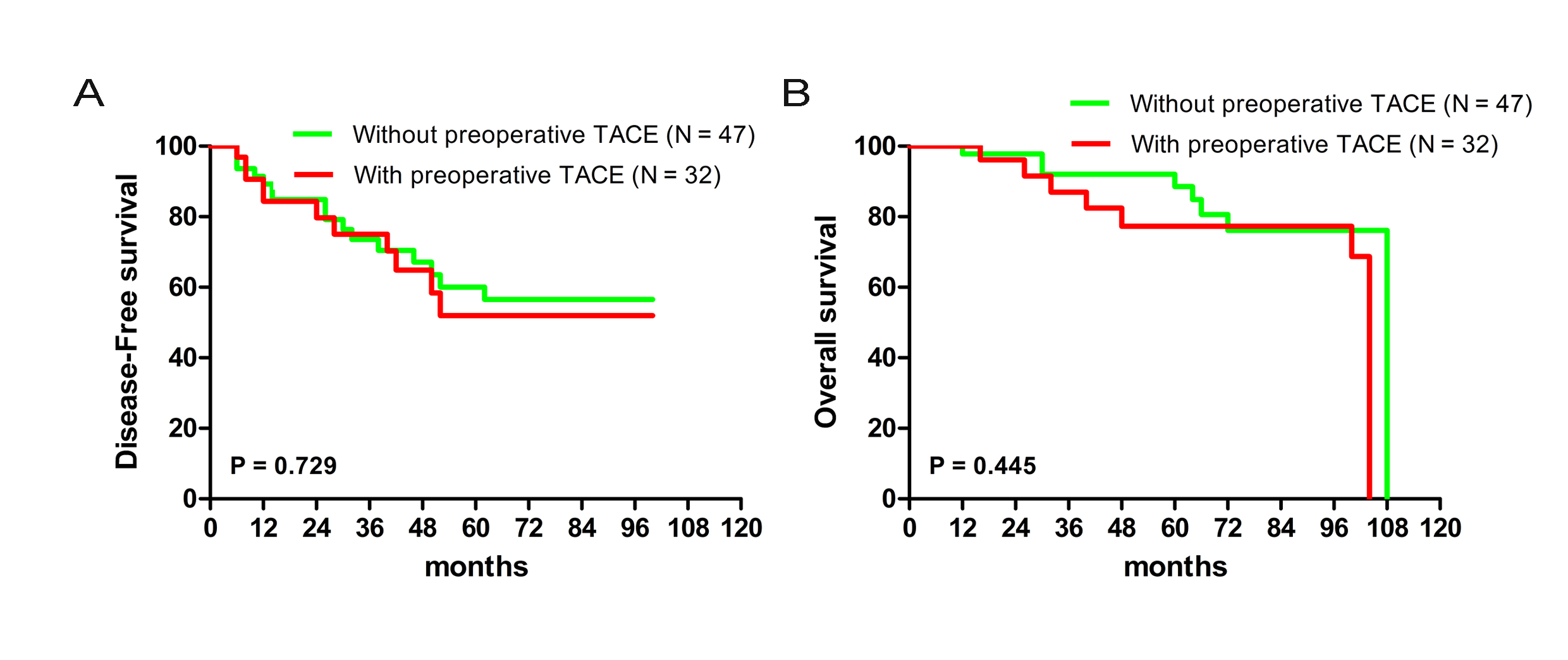

Supplement: Supplementary file 2 — Figure S2 [file CAM4-10-2100-s013.tif]

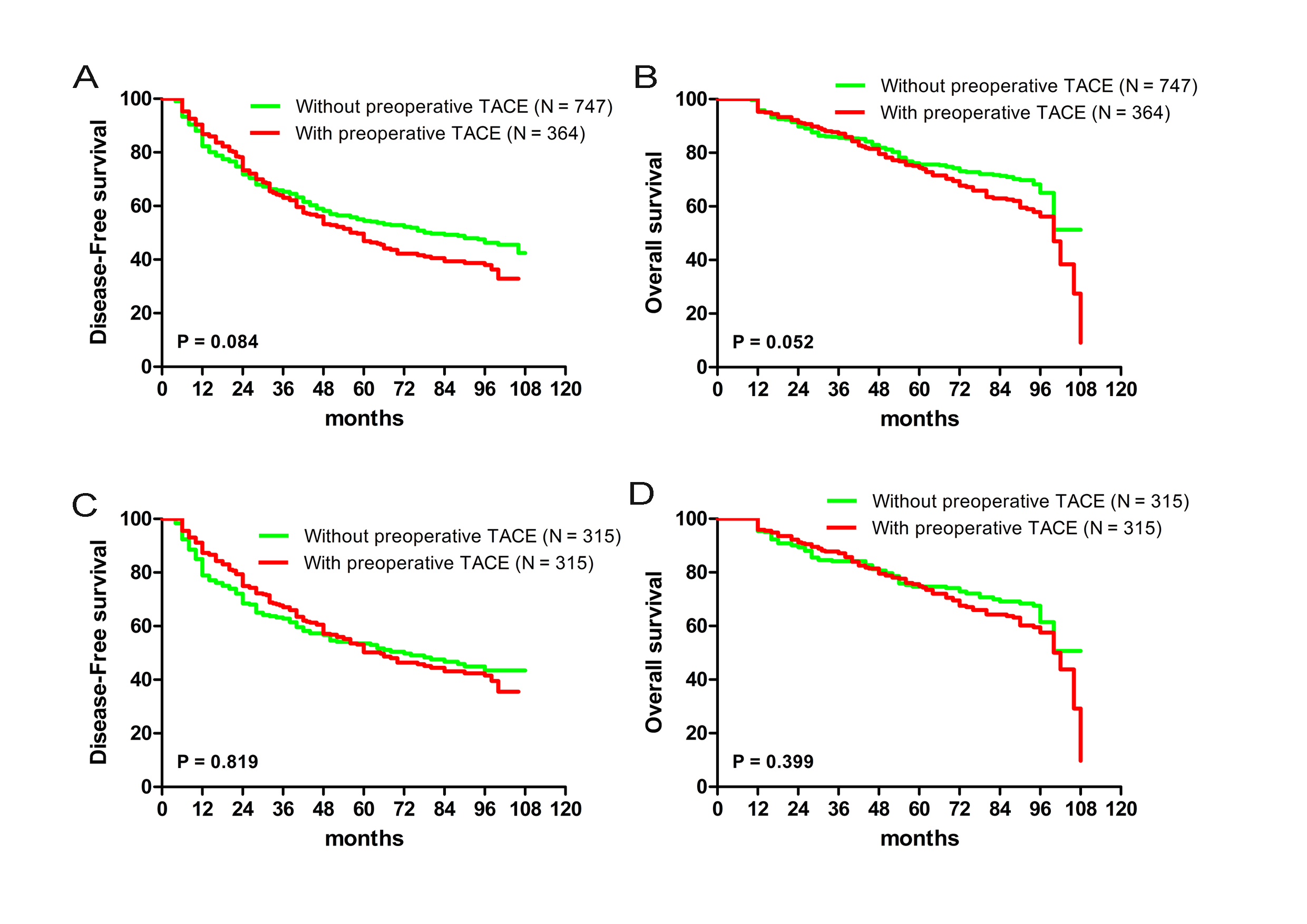

Supplement: Supplementary file 3 — Figure S3 [file CAM4-10-2100-s005.tif]

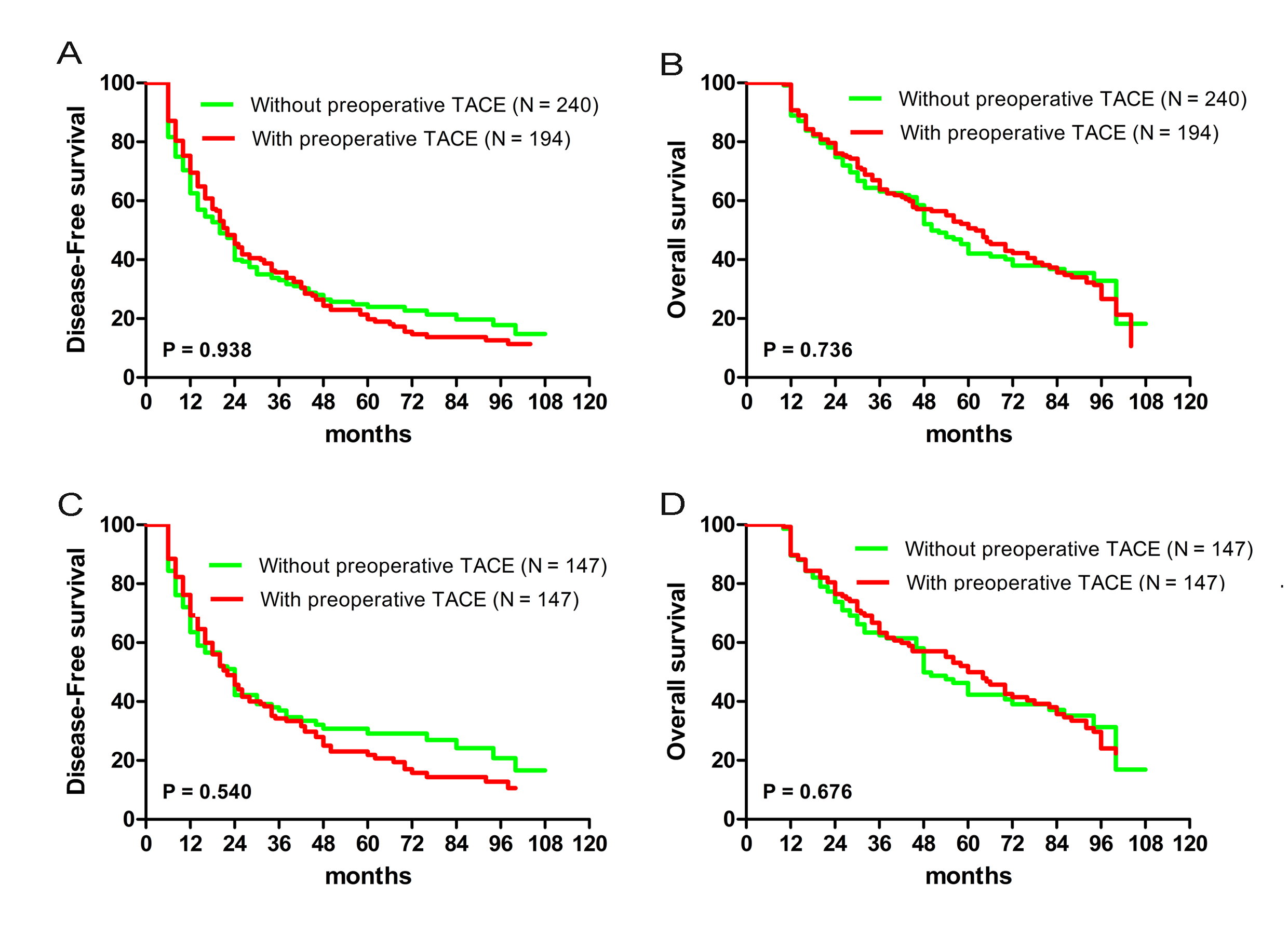

Supplement: Supplementary file 4 — Figure S4 [file CAM4-10-2100-s007.tif]

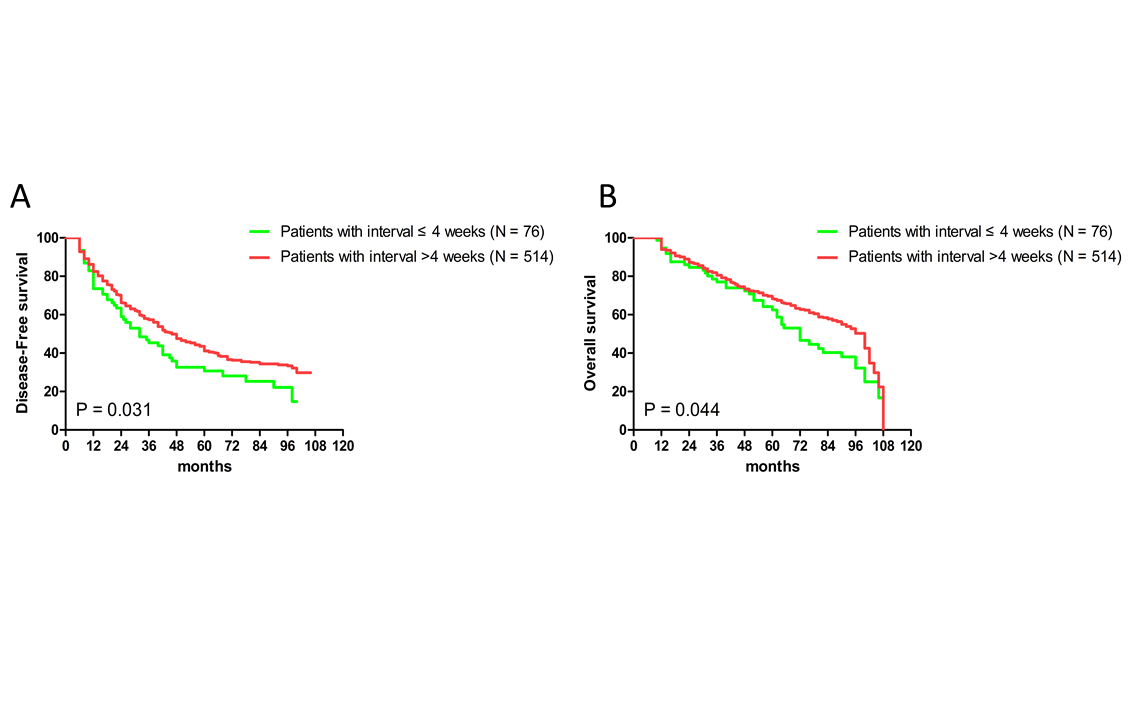

Supplement: Supplementary file 5 — Figure S5 [file CAM4-10-2100-s002.tif]
