# Supplementary material for: Impact of preoperative TACE on incidences of microvascular invasion and long‐term post‐hepatectomy survival in hepatocellular carcinoma patients: A propensity score matching analysis
Source: Cancer Med. 2021 Mar 1;10(6):2100–11. doi: 10.1002/cam4.3814 (PMC7957201; doi:10.1002/cam4.3814)
Supplement: Supplementary file 6 — Table S1 [file CAM4-10-2100-s011.docx]

| Supplemental Table 1. Comparisons of patients’ baseline characteristics between patients with preoperative transarterial chemoembolization (TACE) and without preoperative transarterial chemoembolization (TACE) in BCLC stage 0 | | | |
| --- | --- | --- | --- |
| Variables | With preoperative TACE(N=32) | Without preoperative TACE(N=47) |  |
|  | N (%) | N (%) | P |
| Age,years(Mean±SD) | 53.72±7.32 | 50.19±9.75 | 0.086 |
| Gender |  |  | 0.874 |
| Male | 23(71.87) | 33(70.21) |  |
| Female | 9(28.13) | 14(29.79) |  |
| HbsAg |  |  | 0.720 |
| + | 27(84.37) | 41(87.23) |  |
| - | 5(15.63) | 6(12.77) |  |
| HbeAg |  |  | 0.732 |
| + | 10(31.25) | 13(27.66) |  |
| - | 22(68.75) | 34(72.34) |  |
| HCV Ab |  |  | 1.000 |
| + | 0(0.00) | 0(0) |  |
| - | 32(100) | 47(100) |  |
| HBV DNA |  |  | 0.246 |
| ≥10000IU/ml | 7(21.88) | 16(34.04) |  |
| <10000IU/ml | 25(78.12) | 31(65.96) |  |
| AFP |  |  | 0.592 |
| ≥400ng/ml | 7(21.88) | 8(17.02) |  |
| <400ng/ml | 25(78.12) | 39(82.98) |  |
| TBIL |  |  | 0.354 |
| ≥17umol/L | 9(28.13) | 9(19.15) |  |
| <17umol/L | 23(71.87) | 38(80.85) |  |
| ALB |  |  | 0.986 |
| ≥35g/L | 19(59.38) | 28(59.57) |  |
| <35g/L | 13(40.62) | 19(40.43) |  |
| ALT |  |  | 0.052 |
| ≥44U/L | 18(56.25) | 16(34.04) |  |
| <44U/L | 14(43.75) | 31(65.96) |  |
| PLT |  |  | <0.001 |
| ≥100*10^9/L | 10(31.25) | 38(80.85) |  |
| <100*10^9/L | 22(68.75) | 9(19.15) |  |
| Tumor number |  |  | 1.000 |
| Single | 32(100) | 47(100) |  |
| Multiple | 0(0) | 0(0) |  |
| Liver Cirrhosis |  |  | 0.001 |
| Yes | 24(75.00) | 18(38.30) |  |
| No | 8(25.00) | 29(61.70) |  |
| Max Tumor diameter(Mean±SD) | 1.54±0.33cm | 1.59±0.32cm | 0.453 |
| Tumor capsule |  |  | 0.022 |
| Absent or Partial | 23(71.88) | 43(91.49) |  |
| Complete | 9(28.12) | 4(8.51) |  |
| Tumor margin |  |  | 0.870 |
| Smooth | 27(84.38) | 39(82.98) |  |
| Non-smooth | 5(15.62) | 8(17.02) |  |
| Edmondson Grade |  |  | 0.354 |
| I+II | 9(28.13) | 9(19.15) |  |
| III+IV | 23(71.87) | 38(80.85) |  |
| Satellite Nodules |  |  | 1.000 |
| Presence | 0(0) | 0(0) |  |
| Absence | 32(100) | 47(100) |  |
| Abbreviations: TACE, transcatheter arterial chemoembolization; BCLC, Barcelona Clinic Liver Cancer; HBV, hepatitis B virus; HCV Ab, hepatitis C virus antibody; DNA, deoxyribonucleic acid; TBIL, total bilirubin; ALT, alanine aminotransferase; ALB, albumin; PLT, platelet; AFP, serum alpha-fetoprotein; HBeAg, hepatitis B e antigen; HBsAg, hepatitis B surface antigen; | | | |
|  |  |  |  |
|  |  |  |  |
|  |  |  |  |
|  |  |  |  |
