# Supplementary material for: Impact of preoperative TACE on incidences of microvascular invasion and long‐term post‐hepatectomy survival in hepatocellular carcinoma patients: A propensity score matching analysis
Source: Cancer Med. 2021 Mar 1;10(6):2100–11. doi: 10.1002/cam4.3814 (PMC7957201; doi:10.1002/cam4.3814)
Supplement: Supplementary file 7 — Table S2 [file CAM4-10-2100-s012.docx]

| Supplemental Table 2. Comparisons of patients’ baseline characteristics between patients with preoperative transarterial chemoembolization (TACE) and without preoperative transarterial chemoembolization (TACE) in BCLC stage A before and after propensity score matching (PSM) | | | | | | | | |
| --- | --- | --- | --- | --- | --- | --- | --- | --- |
| The entire cohort | | | |  | The PSM cohort | | | |
| Variables | With preoperative TACE(N=364) | Without preoperative TACE(N=747) |  |  | Variables | With preoperative TACE(N=315) | Without preoperative TACE(N=315) |  |
|  | N (%) | N (%) | P |  |  | N (%) | N (%) | P |
| Age,years(Mean±SD) | 50.84±10.68 | 52.53±10.06 | 0.011 |  | Age,years(Mean±SD) | 51.40±10.43 | 51.92±10.22 | 0.527 |
| Gender |  |  | 0.053 |  | Gender |  |  | 0.358 |
| Male | 321(88.19) | 626(83.80) |  |  | Male | 275(87.30) | 267(84.76) |  |
| Female | 43(11.81) | 121(16.20) |  |  | Female | 40(12.70) | 48(15.24) |  |
| HbsAg |  |  | 0.252 |  | HbsAg |  |  | 0.584 |
| + | 311(85.44) | 618(82.73) |  |  | + | 268(85.08) | 263(83.49) |  |
| - | 53(14.56） | 129(17.27) |  |  | - | 47(14.92) | 52(16.51) |  |
| HbeAg |  |  | 0.203 |  | HbeAg |  |  | 0.162 |
| + | 96(26.37） | 171(22.89) |  |  | + | 83(26.35) | 68(21.59) |  |
| - | 268(73.63） | 576(77.11) |  |  | - | 232(73.65) | 247(78.41) |  |
| HCV Ab |  |  | 0.053 |  | HCV Ab |  |  | 0.737 |
| + | 11(3.02） | 10(1.34) |  |  | + | 5(1.59) | 4(1.27) |  |
| - | 353(96.98） | 737(98.66) |  |  | - | 310(98.41) | 311(98.73) |  |
| HBV DNA |  |  | 0.001 |  | HBV DNA |  |  | 0.245 |
| ≥10000IU/ml | 87(23.90） | 253(33.87) |  |  | ≥10000IU/ml | 79(25.08) | 92(29.21) |  |
| <10000IU/ml | 277(76.10） | 494(66.13) |  |  | <10000IU/ml | 236(74.92) | 223(70.79) |  |
| AFP |  |  | 0.201 |  | AFP |  |  | 0.868 |
| ≥400ng/ml | 133(36.54） | 244(32.66) |  |  | ≥400ng/ml | 115(36.51) | 113(35.87) |  |
| <400ng/ml | 231(63.46） | 503(67.34) |  |  | <400ng/ml | 200(63.49) | 202(64.13) |  |
| TBIL |  |  | <0.001 |  | TBIL |  |  | 0.846 |
| ≥17umol/L | 96(26.37） | 108(14.46) |  |  | ≥17umol/L | 68(21.59) | 66(20.95) |  |
| <17umol/L | 268(73.63） | 639(85.54) |  |  | <17umol/L | 247(78.41) | 249(79.05) |  |
| ALB |  |  | <0.001 |  | ALB |  |  | 0.871 |
| ≥35g/L | 195(53.57） | 537(71.89) |  |  | ≥35g/L | 185(58.73) | 187(59.37) |  |
| <35g/L | 169(46.43） | 210(28.11) |  |  | <35g/L | 130(41.27) | 128(40.63) |  |
| ALT |  |  | 0.001 |  | ALT |  |  | 0.337 |
| ≥44U/L | 172(47.25） | 277(37.08) |  |  | ≥44U/L | 136(43.17) | 148(46.98) |  |
| <44U/L | 192(52.75） | 470(62.92) |  |  | <44U/L | 179(56.83) | 167(53.02) |  |
| PLT |  |  | 0.002 |  | PLT |  |  | 0.702 |
| ≥100*10^9/L | 277(76.10） | 627(83.94) |  |  | ≥100*10^9/L | 247(78.41) | 243(77.14) |  |
| <100*10^9/L | 87(23.90) | 120(16.06) |  |  | <100*10^9/L | 68(21.59) | 72(22.86) |  |
| Tumor number |  |  | 0.093 |  | Tumor number |  |  | 1.000 |
| Single | 346(95.05) | 725(97.05) |  |  | Single | 298(94.60) | 298(94.60) |  |
| Multiple | 18(4.95) | 22(2.95) |  |  | Multiple | 17(5.40) | 17(5.40) |  |
| Liver Cirrhosis |  |  | <0.001 |  | Liver Cirrhosis |  |  | 0.364 |
| Yes | 156(42.86) | 196(26.24) |  |  | Yes | 122(38.73) | 111(35.24) |  |
| No | 208(57.14) | 551(73.76) |  |  | No | 193(61.27) | 204(64.76) |  |
| Max Tumor diameter(Mean±SD) | 6.97±4.30cm | 6.02±3.88cm | <0.001 |  | Max Tumor diameter(Mean±SD) | 6.65±4.06cm | 6.57±4.32cm | 0.808 |
| Tumor capsule |  |  | 0.003 |  | Tumor capsule |  |  | 0.135 |
| Absent or Partial | 277(76.10) | 624(83.53) |  |  | Absent or Partial | 248(78.73) | 232(73.65) |  |
| Complete | 87(23.90) | 123(16.47) |  |  | Complete | 67(21.27) | 83(26.35) |  |
| Tumor margin |  |  | 0.001 |  | Tumor margin |  |  | 0.387 |
| Smooth | 315(86.54) | 584(78.18) |  |  | Smooth | 268(85.08) | 260(82.54) |  |
| Non-smooth | 49(13.46) | 163(21.82) |  |  | Non-smooth | 47(14.92) | 55(17.46) |  |
| Edmondson Grade |  |  | 0.949 |  | Edmondson Grade |  |  | 0.281 |
| I+II | 41(11.26) | 95(12.72) |  |  | I+II | 37(11.75) | 47(14.92) |  |
| III+IV | 285(78.30) | 652(87.28) |  |  | III+IV | 272(86.35) | 268(85.08) |  |
| Satellite Nodules |  |  | <0.001 |  | Satellite Nodules |  |  | 0.934 |
| Presence | 117(32.14) | 337(45.11) |  |  | Presence | 111(35.24) | 110(34.92) |  |
| Absence | 247(67.86) | 410(54.89) |  |  | Absence | 204(64.76) | 205(65.08) |  |
| Abbreviations: TACE, transcatheter arterial chemoembolization; HBV, hepatitis B virus; Barcelona Clinic Liver Cancer HCV Ab, hepatitis C virus antibody; DNA, deoxyribonucleic acid; TBIL, total bilirubin; ALT, alanine aminotransferase; ALB, albumin; PLT, platelet; AFP, serum alpha-fetoprotein; HBeAg, hepatitis B e antigen; HBsAg, hepatitis B surface antigen; PSM, Propensity score matching | | | | | | | | |
|  |  |  |  |  |  |  |  |  |
|  |  |  |  |  |  |  |  |  |
