# Supplementary material for: Impact of preoperative TACE on incidences of microvascular invasion and long‐term post‐hepatectomy survival in hepatocellular carcinoma patients: A propensity score matching analysis
Source: Cancer Med. 2021 Mar 1;10(6):2100–11. doi: 10.1002/cam4.3814 (PMC7957201; doi:10.1002/cam4.3814)
Supplement: Supplementary file 8 — Table S3 [file CAM4-10-2100-s006.docx]

| Supplemental Table 3. Comparisons of patients’ baseline characteristics between patients with preoperative transarterial chemoembolization (TACE) and without preoperative transarterial chemoembolization (TACE) in BCLC stage B before and after propensity score matching (PSM) | | | | | | | | | |
| --- | --- | --- | --- | --- | --- | --- | --- | --- | --- |
| The entire cohort | | | |  | The PSM cohort | | | | |
| Variables | With preoperative TACE(N=194) | Without preoperative TACE(N=240) |  |  | Variables | With preoperative TACE(N=147) | | Without preoperative TACE(N=147) |  |
|  | N (%) | N (%) | P |  |  | N (%) | | N (%) | P |
| Age,years(Mean±SD) | 48.46±10.05 | 50.50±10.01 |  |  | Age,years(Mean±SD) | 48.97±9.93 | 50.10±9.95 | | 0.331 |
| Gender |  |  | 0.234 |  | Gender |  |  | | 0.708 |
| Male | 176(90.72) | 209(87.08) |  |  | Male | 132(89.80) | 130(88.44) | |  |
| Female | 18(9.28) | 31(12.92) |  |  | Female | 15(10.20) | 17(11.56) | |  |
| HbsAg |  |  | 0.402 |  | HbsAg |  |  | | 0.356 |
| + | 174(89.69) | 209(87.08) |  |  | + | 133(90.48) | 128(87.07) | |  |
| - | 20(10.31) | 31(12.92) |  |  | - | 14(9.52) | 19(12.93) | |  |
| HbeAg |  |  | 0.513 |  | HbeAg |  |  | | 1.000 |
| + | 55(28.35) | 75(31.25) |  |  | + | 44(29.93) | 44(29.93) | |  |
| - | 139(71.65) | 165(68.75) |  |  | - | 103(70.07) | 103(70.07) | |  |
| HCV Ab |  |  | 0.387 |  | HCV Ab |  |  | | 0.562 |
| + | 2(1.03) | 5(2.08) |  |  | + | 1(0.68) | 2(1.36) | |  |
| - | 192(98.97) | 235(97.92) |  |  | - | 146(99.32) | 145(98.64) | |  |
| HBV DNA |  |  | 0.059 |  | HBV DNA |  |  | | 0.802 |
| ≥10000IU/ml | 50(25.77) | 82(34.17) |  |  | ≥10000IU/ml | 45(30.61) | 47(31.97) | |  |
| <10000IU/ml | 144(74.23) | 158(65.83) |  |  | <10000IU/ml | 102(69.39) | 100(68.03) | |  |
| AFP |  |  | 0.955 |  | AFP |  |  | | 0.555 |
| ≥400ng/ml | 90(46.39) | 112(46.67) |  |  | ≥400ng/ml | 64(43.54) | 59(40.14) | |  |
| <400ng/ml | 104(53.61) | 128(53.33) |  |  | <400ng/ml | 83(56.46) | 88(59.86) | |  |
| TBIL |  |  | <0.001 |  | TBIL |  |  | | 0.872 |
| ≥17umol/L | 57(29.38) | 25(10.42) |  |  | ≥17umol/L | 23(15.65) | 22(14.97) | |  |
| <17umol/L | 137(70.62) | 215(89.58) |  |  | <17umol/L | 124(84.35) | 125(85.03) | |  |
| ALB |  |  | 0.095 |  | ALB |  |  | | 0.548 |
| ≥35g/L | 105(54.12) | 149(62.08) |  |  | ≥35g/L | 89(60.54) | 94(63.95) | |  |
| <35g/L | 89(45.88) | 91(37.92) |  |  | <35g/L | 58(39.46) | 53(36.05) | |  |
| ALT |  |  | 0.105 |  | ALT |  |  | | 0.724 |
| ≥44U/L | 91(46.91) | 94(39.17) |  |  | ≥44U/L | 64(43.53) | 61(41.50) | |  |
| <44U/L | 103(53.09) | 146(60.83) |  |  | <44U/L | 83(56.47) | 86(58.50) | |  |
| PLT |  |  | 0.202 |  | PLT |  |  | | 0.670 |
| ≥100*10^9/L | 147(75.77) | 194(80.83) |  |  | ≥100*10^9/L | 114(77.55) | 117(79.59) | |  |
| <100*10^9/L | 47(24.25) | 46(19.17) |  |  | <100*10^9/L | 33(22.45) | 30(20.41) | |  |
| Tumor number |  |  | 0.094 |  | Tumor number |  |  | | 0.595 |
| Single | 48(24.74) | 77(32.08) |  |  | Single | 40(27.21) | 36(24.49) | |  |
| Multiple | 146(75.26) | 163(67.92) |  |  | Multiple | 107(72.79) | 111(75.51) | |  |
| Liver Cirrhosis |  |  | <0.001 |  | Liver Cirrhosis |  |  | | 0.283 |
| Yes | 93(47.94) | 69(28.75) |  |  | Yes | 62(42.18) | 53(36.05) | |  |
| No | 101(52.06) | 171(71.25) |  |  | No | 85(57.82) | 94(63.95) | |  |
| Max Tumor diameter(Mean±SD) | 8.81±4.90cm | 8.60±4.39cm | 0.648 |  | Max Tumor diameter(Mean±SD) | 8.62±4.86cm | 8.63±4.38cm | | 0.980 |
| Tumor capsule |  |  | 0.031 |  | Tumor capsule |  |  | | 0.607 |
| Absent or Partial | 163(84.02) | 218(90.83) |  |  | Absent or Partial | 126(85.71) | 129(87.76) | |  |
| Complete | 31(15.98) | 22(9.17) |  |  | Complete | 21(14.29) | 18(12.24) | |  |
| Tumor margin |  |  | 0.051 |  | Tumor margin |  |  | | 0.802 |
| Smooth | 137(70.62) | 148(61.67) |  |  | Smooth | 100(68.03) | 102(69.39) | |  |
| Non-smooth | 57(29.38) | 92(38.33) |  |  | Non-smooth | 47(31.97) | 45(30.61) | |  |
| Edmondson Grade |  |  | 0.573 |  | Edmondson Grade |  |  | | 0.769 |
| I+II | 11(5.67) | 11(4.58) |  |  | I+II | 9(6.12) | 8(5.44) | |  |
| III+IV | 179(92.27) | 229(95.42) |  |  | III+IV | 135(91.84) | 139(94.56) | |  |
| Satellite Nodules |  |  | 0.254 |  | Satellite Nodules |  |  | | 0.553 |
| Presence | 156(80.41) | 203(84.58) |  |  | Presence | 117(79.59) | 121(82.31) | |  |
| Absence | 38(19.59) | 37(15.42) |  |  | Absence | 30(20.40) | 26(17.69) | |  |
| Abbreviations: TACE, transcatheter arterial chemoembolization; BCLC, Barcelona Clinic Liver Cancer; HBV, hepatitis B virus; HCV Ab, hepatitis C virus antibody; DNA, deoxyribonucleic acid; TBIL, total bilirubin; ALT, alanine aminotransferase; ALB, albumin; PLT, platelet; AFP, serum alpha-fetoprotein; HBeAg, hepatitis B e antigen; HBsAg, hepatitis B surface antigen; PSM, Propensity score matching | | | | | | | | | |
|  |  |  |  |  |  |  |  |  |  |
|  |  |  |  |  |  |  |  |  |  |
