# Supplementary material for: Impact of preoperative TACE on incidences of microvascular invasion and long‐term post‐hepatectomy survival in hepatocellular carcinoma patients: A propensity score matching analysis
Source: Cancer Med. 2021 Mar 1;10(6):2100–11. doi: 10.1002/cam4.3814 (PMC7957201; doi:10.1002/cam4.3814)
Supplement: Supplementary file 9 — Table S4 [file CAM4-10-2100-s010.docx]

| Supplemental Table 4. Univariable and multivariable Cox regression analyses with factors of Disease-Free survival after curative resection of hepatocellular carcinoma in the entire cohort | | | | | | |
| --- | --- | --- | --- | --- | --- | --- |
|  | Univariable | |  | Multivariable | |  |
| Variable | HR (95% CI) | P |  | HR (95% CI) | P |  |
| Preoperative TACE(Yes vs No) | 1.176(1.025-1.351) | 0.021 |  | 1.104(0.957-1.275) | 0.174 |  |
| Tumor number(Multiple vs Single) | 1.557(1.332-1.820) | <0.001 |  | 1.367(1.144-1.635) | 0.001 |  |
| Tumor Size(≥5cm vs <5cm) | 2.249(1.947-2.599) | <0.001 |  | 1.952(1.681-2.268) | <0.001 |  |
| Satellite Nodules(Presence vs Absence) | 1.492(1.300-1.712) | <0.001 |  | 0.964(0.809-1.148) | 0.678 |  |
| Edmondson Grade(III+IV vs I+II) | 1.681(1.324-2.135) | <0.001 |  | 1.222(0.954-1.565) | 0.112 |  |
| Tumor capsule(Non-complete vs Complete) | 1.366(1.135-1.644) | 0.001 |  | 1.212(0.996-1.476) | 0.055 |  |
| Liver Cirrhosis(Yes vs No) | 1.077(0.933-1.243) | 0.309 |  | - | - |  |
| Age(≥60 vs <60) | 0.832(0.706-0.980) | 0.027 |  | 1.027(0.868-1.216) | 0.754 |  |
| Gender(Male vs Female) | 1.095(0.895-1.339) | 0.378 |  | - | - |  |
| Tumor margin(Non-smooth vs Smooth) | 1.593(1.364-1.860) | <0.001 |  | 1.124(0.941-1.342) | 0.199 |  |
| HCV Ab(Positive vs Negative) | 0.798(0.451-1.411) | 0.473 |  | - | - |  |
| HBV DNA(≥10000IU/ml VS <10000IU/ml) | 1.057(0.912-1.226) | 0.458 |  | - | - |  |
| TBIL(≥17umol/L vs <17umol/L) | 1.023(0.861-1.216) | 0.795 |  | - | - |  |
| ALT(≥44U/L vs <44U/L) | 1.120(0.977-1.285) | 0.105 |  | - | - |  |
| ALB(<35g/L vs ≥35g/L) | 1.168(1.016-1.342) | 0.029 |  | 1.133(0.983-1.306) | 0.084 |  |
| PLT(<100*10^9/L vs ≥100*10^9/L) | 1.011(0.855-1.196) | 0.894 |  | - | - |  |
| AFP(≥400ng/ml vs <400ng/ml) | 1.723(1.503-1.977) | <0.001 |  | 1.357(1.175-1.566) | <0.001 |  |
| HbeAg(Positive vs Negative) | 1.171(1.007-1.363) | 0.041 |  | 1.185(1.016-1.382) | 0.031 |  |
| HbsAg(Positive vs Negative) | 1.091(0.900-1.323) | 0.377 |  | - | - |  |
| MVI(Positive vs Negative) | 1.893(1.650-2.171) | <0.001 |  | 1.571(1.339-1.843) | <0.001 |  |
| Abbreviations: TACE, transcatheter arterial chemoembolization; HBV, hepatitis B virus; HCV Ab, hepatitis C virus antibody; DNA, deoxyribonucleic acid; TBIL, total bilirubin; ALT, alanine aminotransferase; ALB, albumin; PLT, platelet; AFP, serum alpha-fetoprotein; HBeAg, hepatitis B e antigen; HBsAg, hepatitis B surface antigen; MVI, microvascular invasion; 95% CI, 95 Percent confidence interval; HR, hazard ratio | | | | | |  |
