# Supplementary material for: Impact of preoperative TACE on incidences of microvascular invasion and long‐term post‐hepatectomy survival in hepatocellular carcinoma patients: A propensity score matching analysis
Source: Cancer Med. 2021 Mar 1;10(6):2100–11. doi: 10.1002/cam4.3814 (PMC7957201; doi:10.1002/cam4.3814)
Supplement: Supplementary file 10 — Table S5 [file CAM4-10-2100-s003.docx]

| Supplemental Table 5. Univariable and multivariable Cox regression analyses with factors of Overall Survival after curative resection of hepatocellular carcinoma in the entire cohort | | | | | |
| --- | --- | --- | --- | --- | --- |
|  | Univariable | |  | Multivariable | |
| Variable | HR (95% CI) | P |  | HR (95% CI) | P |
| Preoperative TACE(Yes vs No) | 1.191(1.010-1.405) | 0.038 |  | 1.126(0.948-1.336) | 0.176 |
| Tumor number(Multiple vs Single) | 1.497(1.242-1.803) | <0.001 |  | 1.201(0.967-1.491) | 0.098 |
| Tumor Size(≥5cm vs <5cm) | 2.788(2.322-3.348) | <0.001 |  | 2.284(1.893-2.757) | <0.001 |
| Satellite Nodules(Presence vs Absence) | 1.677(1.421-1.979) | <0.001 |  | 1.059(0.857-1.309) | 0.596 |
| Edmondson Grade(III+IV vs I+II) | 2.654(1.885-3.737) | <0.001 |  | 1.736(1.223-2.464) | 0.002 |
| Tumor capsule(Non-complete vs Complete) | 1.581(1.257-1.990) | <0.001 |  | 1.245(0.976-1.589) | 0.078 |
| Liver Cirrhosis(Yes vs No) | 0.928(0.779-1.105) | 0.399 |  | - | - |
| Age(≥60 vs <60) | 0.812(0.663-0.996) | 0.045 |  | - | - |
| Gender(Male vs Female) | 0.986(0.775-1.253) | 0.907 |  | - | - |
| Tumor margin(Non-smooth vs Smooth) | 1.793(1.492-2.155) | <0.001 |  | 1.142(0.927-1.405) | 0.211 |
| HCV Ab(Positive vs Negative) | 0.646(0.306-1.361) | 0.250 |  | - | - |
| HBV DNA(≥10000IU/ml VS <10000IU/ml) | 1.004(0.838-1.202) | 0.968 |  | - | - |
| TBIL(≥17umol/L vs <17umol/L) | 1.039(0.847-1.276) | 0.712 |  | - | - |
| ALT(≥44U/L vs <44U/L) | 1.028(0.871-1.214) | 0.740 |  | - | - |
| ALB(<35g/L vs ≥35g/L) | 1.193(1.010-1.409) | 0.038 |  | 1.171(0.988-1.388) | 0.069 |
| PLT(<100*10^9/L vs ≥100*10^9/L) | 1.001(0.819-1.223) | 0.995 |  | - | - |
| AFP(≥400ng/ml vs <400ng/ml) | 1.961(1.664-2.311) | <0.001 |  | 1.431(1.207-1.696) | <0.001 |
| HbeAg(Positive vs Negative) | 1.164(0.970-1.397) | 0.102 |  | - | - |
| HbsAg(Positive vs Negative) | 1.002(0.800-1.254) | 0.989 |  | - | - |
| MVI(Positive vs Negative) | 2.313(1.959-2.730) | <0.001 |  | 1.743(1.438-2.113) | <0.001 |
| Abbreviations: TACE, transcatheter arterial chemoembolization; HBV, hepatitis B virus; HCV Ab, hepatitis C virus antibody; DNA, deoxyribonucleic acid; TBIL, total bilirubin; ALT, alanine aminotransferase; ALB, albumin; PLT, platelet; AFP, serum alpha-fetoprotein; HBeAg, hepatitis B e antigen; HBsAg, hepatitis B surface antigen; MVI, microvascular invasion; 95% CI, 95 Percent confidence interval; HR, hazard ratio | | | | | |
