# Supplementary material for: Impact of preoperative TACE on incidences of microvascular invasion and long‐term post‐hepatectomy survival in hepatocellular carcinoma patients: A propensity score matching analysis
Source: Cancer Med. 2021 Mar 1;10(6):2100–11. doi: 10.1002/cam4.3814 (PMC7957201; doi:10.1002/cam4.3814)
Supplement: Supplementary file 11 — Table S6 [file CAM4-10-2100-s001.docx]

| Supplemental Table 6. Univariable and multivariable Cox regression analyses with factors of Disease-Free survival after curative resection of hepatocellular carcinoma in the propensity matched cohort | | | | | |
| --- | --- | --- | --- | --- | --- |
|  | Univariable | |  | Multivariable | |
| Variable | HR (95% CI) | P |  | HR (95% CI) | P |
| Preoperative TACE(Yes vs No) | 1.098(0.923-1.307) | 0.292 |  | - | - |
| Tumor number(Multiple vs Single) | 1.308(1.072-1.595) | 0.008 |  | 1.237(0.987-1.551) | 0.065 |
| Tumor Size(≥5cm vs <5cm) | 2.288(1.902-2.753) | <0.001 |  | 2.016(1.670-2.432) | <0.001 |
| Satellite Nodules(Presence vs Absence) | 1.484(1.246-1.768) | <0.001 |  | 0.955(0.763-1.196) | 0.690 |
| Edmondson Grade(III+IV vs I+II) | 1.667(1.254-2.217) | <0.001 |  | 1.227(0.914-1.647) | 0.172 |
| Tumor capsule(Non-complete vs Complete) | 1.454(1.161-1.822) | 0.001 |  | 1.191(0.936-1.516) | 0.155 |
| Liver Cirrhosis(Yes vs No) | 1.065(0.892-1.273) | 0.486 |  | - | - |
| Age(≥60 vs <60) | 0.833(0.674-1.029) | 0.090 |  | - | - |
| Gender(Male vs Female) | 1.004(0.780-1.292) | 0.973 |  | - | - |
| Tumor margin(Non-smooth vs Smooth) | 1.772(1.444-2.174) | <0.001 |  | 1.340(1.069-1.681) | 0.011 |
| HCV Ab(Positive vs Negative) | 0.841(0.418-1.691) | 0.627 |  | - | - |
| HBV DNA(≥10000IU/ml VS <10000IU/ml) | 1.128(0.933-1.365) | 0.213 |  | - | - |
| TBIL(≥17umol/L vs <17umol/L) | 0.935(0.753-1.162) | 0.544 |  | - | - |
| ALT(≥44U/L vs <44U/L) | 1.012(0.850-1.204) | 0.897 |  | - | - |
| ALB(<35g/L vs ≥35g/L) | 1.203(1.101-1.433) | 0.038 |  | 1.206(1.010-1.439) | 0.038 |
| PLT(<100*10^9/L vs ≥100*10^9/L) | 0.961(0.784-1.177) | 0.699 |  | - | - |
| AFP(≥400ng/ml vs <400ng/ml) | 1.670(1.403-1.988) | <0.001 |  | 1.368(1.143-1.639) | 0.001 |
| HbeAg(Positive vs Negative) | 1.126(0.926-1.369) | 0.236 |  | - | - |
| HbsAg(Positive vs Negative) | 1.009(0.792-1.285) | 0.944 |  | - | - |
| MVI(Positive vs Negative) | 1.980(1.660-2.360) | <0.001 |  | 1.586(1.302-1.931) | <0.001 |
| Abbreviations: TACE, transcatheter arterial chemoembolization; HBV, hepatitis B virus; HCV Ab, hepatitis C virus antibody; DNA, deoxyribonucleic acid; TBIL, total bilirubin; ALT, alanine aminotransferase; ALB, albumin; PLT, platelet; AFP, serum alpha-fetoprotein; HBeAg, hepatitis B e antigen; HBsAg, hepatitis B surface antigen; MVI, microvascular invasion; 95% CI, 95 Percent confidence interval; HR, hazard ratio | | | | | |
