# Supplementary material for: Impact of preoperative TACE on incidences of microvascular invasion and long‐term post‐hepatectomy survival in hepatocellular carcinoma patients: A propensity score matching analysis
Source: Cancer Med. 2021 Mar 1;10(6):2100–11. doi: 10.1002/cam4.3814 (PMC7957201; doi:10.1002/cam4.3814)
Supplement: Supplementary file 12 — Table S7 [file CAM4-10-2100-s008.docx]

| Supplemental Table 7. Univariable and multivariable Cox regression analyses with factors of Overall survival after curative resection of hepatocellular carcinoma in the propensity matched cohort | | | | | | |
| --- | --- | --- | --- | --- | --- | --- |
|  | Univariable | |  | Multivariable | |  |
| Variable | HR (95% CI) | P |  | HR (95% CI) | P |  |
| Preoperative TACE(Yes vs No) | 1.140(0.925-1.404) | 0.219 |  | - | - |  |
| Tumor number(Multiple vs Single) | 1.282(1.013-1.622) | 0.039 |  | 0.993(0.761-1.296) | 0.959 |  |
| Tumor Size(≥5cm vs <5cm) | 2.682(2.139-3.364) | <0.001 |  | 2.294(1.824-2.884) | <0.001 |  |
| Satellite Nodules(Presence vs Absence) | 1.855(1.507-2.283) | <0.001 |  | 1.267(0.976-1.643) | 0.075 |  |
| Edmondson Grade(III+IV vs I+II) | 2.853(1.899-4.286) | <0.001 |  | 1.963(1.294-2.979) | 0.002 |  |
| Tumor capsule(Non-complete vs Complete) | 1.790(1.351-2.371) | <0.001 |  | 1.230(0.911-1.660) | 0.176 |  |
| Liver Cirrhosis(Yes vs No) | 0.926(0.749-1.146) | 0.480 |  | - | - |  |
| Age(≥60 vs <60) | 0.881(0.686-1.132) | 0.322 |  | - | - |  |
| Gender(Male vs Female) | 0.945(0.701-1.274) | 0.710 |  | - | - |  |
| Tumor margin(Non-smooth vs Smooth) | 1.895(1.493-2.407) | <0.001 |  | 1.252(0.966-1.623) | 0.090 |  |
| HCV Ab(Positive vs Negative) | 0.967(0.432-2.168) | 0.936 |  | - | - |  |
| HBV DNA(≥10000IU/ml VS <10000IU/ml) | 1.017(0.810-1.278) | 0.882 |  | - | - |  |
| TBIL(≥17umol/L vs <17umol/L) | 1.000(0.777-1.288) | 0.999 |  | - | - |  |
| ALT(≥44U/L vs <44U/L) | 0.939(0.764-1.155) | 0.553 |  | - | - |  |
| ALB(<35g/L vs ≥35g/L) | 1.074(0.872-1.321) | 0.502 |  | - | - |  |
| PLT(<100*10^9/L vs ≥100*10^9/L) | 0.912(0.716-1.162) | 0.457 |  | - | - |  |
| AFP(≥400ng/ml vs <400ng/ml) | 1.868(1.521-2.294) | <0.001 |  | 1.408(1.138-1.741) | 0.002 |  |
| HbeAg(Positive vs Negative) | 1.096(0.869-1.382) | 0.440 |  | - | - |  |
| HbsAg(Positive vs Negative) | 0.916(0.697-1.204) | 0.531 |  | - | - |  |
| MVI(Positive vs Negative) | 2.417(1.964-2.974) | <0.001 |  | 1.666(1.320-2.103) | <0.001 |  |
| Abbreviations: TACE, transcatheter arterial chemoembolization; HBV, hepatitis B virus; HCV Ab, hepatitis C virus antibody; DNA, deoxyribonucleic acid; TBIL, total bilirubin; ALT, alanine aminotransferase; ALB, albumin; PLT, platelet; AFP, serum alpha-fetoprotein; HBeAg, hepatitis B e antigen; HBsAg, hepatitis B surface antigen; MVI, microvascular invasion; 95% CI, 95 Percent confidence interval; HR, hazard ratio | | | | | |  |
