# Supplementary material for: Impact of preoperative TACE on incidences of microvascular invasion and long‐term post‐hepatectomy survival in hepatocellular carcinoma patients: A propensity score matching analysis
Source: Cancer Med. 2021 Mar 1;10(6):2100–11. doi: 10.1002/cam4.3814 (PMC7957201; doi:10.1002/cam4.3814)
Supplement: Supplementary file 13 — Table S7 [file CAM4-10-2100-s004.docx]

**Supplemental Figure Legends**

Supplemental Figure. 1. Selection of the study population. EHBH Eastern Hepatobiliary Surgery Hospital, HCC hepatocellular carcinoma, TACE transarterial chemoembolization.

Supplemental Figure. 2. Survival curves of HCC patients with preoperative TACE and without preoperative TACE in Barcelona Clinic Liver Cancer (BCLC) stage 0. (A) The cumulative Disease-free survival (DFS) curve of HCC patients with preoperative TACE and without preoperative TACE in BCLC stage 0 (P = 0.729). (B) The cumulative overall survival (OS) curve of HCC patients with preoperative TACE and without preoperative TACE in BCLC stage 0 (P = 0.445).

Supplemental Figure. 3. Survival curves of HCC patients with preoperative TACE and without preoperative TACE in BCLC stage A. (A) The cumulative Disease-free survival (DFS) curve of patients with preoperative TACE and without preoperative TACE in BCLC stage A before PSM (P = 0.084). (B) The cumulative overall survival (OS) curve of patients with preoperative TACE and without preoperative TACE in BCLC stage A before PSM (P = 0.052). (C) (A) The cumulative Disease-free survival (DFS) curve of patients with preoperative TACE and without preoperative TACE in BCLC stage A after PSM (P = 0.819). (D) The cumulative overall survival (OS) curve of patients with preoperative TACE and without preoperative TACE in BCLC stage A after PSM (P = 0.399).

Supplemental Figure. 4. Survival curves of HCC patients with preoperative TACE and without preoperative TACE in BCLC stage B. (A) The cumulative Disease-free survival (DFS) curve of patients with preoperative TACE and without preoperative TACE in BCLC stage B before PSM (P = 0.938). (B) The cumulative overall survival (OS) curve of patients with preoperative TACE and without preoperative TACE in BCLC stage B before PSM (P = 0.736). (C) (A) The cumulative Disease-free survival (DFS) curve of patients with preoperative TACE and without preoperative TACE in BCLC stage B after PSM (P = 0.540). (D) The cumulative overall survival (OS) curve of patients with preoperative TACE and without preoperative TACE in BCLC stage B after PSM (P = 0.676).

Supplemental Figure. 5. Survival analysis of patients with different interval between the first TACE and liver resection. (A) The cumulative Disease-free survival (DFS) curve of HCC patients with interval ≤4 weeks and >4 weeks (P = 0.031). (B) The cumulative overall survival (OS) curve of HCC patients with interval ≤4 weeks and >4 weeks (P = 0.044).

Abbreviations: HCC, hepatocellular carcinoma; TACE, transcatheter arterial chemoembolization; MVI, microvascular invasion; PSM, Propensity score matching; EHBH, Eastern Hepatobiliary Surgery Hospital; DFS, disease-free survival; OS, overall survival; BCLC, Barcelona Clinic Liver Cancer; CT, computed tomography; MRI, magnetic resonance imaging; HBsAg, hepatitis B surface antigen; HBeAg, hepatitis B e antigen; HBV, hepatitis B virus; HCV, hepatitis C virus; HCV Ab, hepatitis C virus antibody; DNA, deoxyribonucleic acid; RNA, ribonucleic acid; AFP, serum alpha-fetoprotein; CEA, carcinoembryonic antigen; CA 19-9, carbohydrate antigen 19-9; ALT, alanine aminotransferase; TBIL, total bilirubin; ALB, albumin; PLT, platelet; PRFA, percutaneous radiofrequency ablation; PEI, percutaneous ethanol injection; PVE, portal vein embolization; 95% CI, 95 percent confidence interval; OR, odds ratio; HR, hazard ratio
